# Predictors of institutional delivery in Sodo town, Southern Ethiopia

**DOI:** 10.4102/phcfm.v5i1.544

**Published:** 2013-11-22

**Authors:** Feleke Hailemichael, Mirkuzie Woldie, Fikru Tafese

**Affiliations:** 1 School of Public Health, Wolaita Sodo University, Ethiopia; 2 Department of Health Services Management, Jimma University, Ethiopia

## Abstract

**Background:**

Women are more liable to die during or following delivery than during pregnancy but use of both delivery services and post-partum care is low.

**Objective:**

To find out the prevalence and predictors of institutional delivery in Wolaita Sodo (Sodo) town, southern Ethiopia.

**Methods:**

A cross-sectional study was used to look at 844 women who had given birth in the previous five years in Sodo town. The study employed a multistage-sampling scheme. Codes were given for all identified women in selected *kebeles* (neighbourhoods) and a simple random-sampling technique was used after generating random numbers using the Statistical Package for Social Sciences (SPSS). SPSS was then used to carry out binary- and multiple logistic regressions. A 95% CI for the odds ratio was applied to judge the presence of relationships between variables.

**Results:**

The prevalence of institutional delivery-service utilisation in Sodo town was 62.2%. Husband educational status, parity, number of antenatal clinic visits, perceived quality of care and knowledge regarding pregnancy danger signs were independent predictors of utilisation of institutional delivery services.

**Conclusion:**

Institutional delivery service utilisation in Sodo town was much higher than the national figure. Findings in this study showed that promotion of antenatal care, involvement of men in maternal healthcare, provision of health education regarding the danger signs of pregnancy and improvement of service quality are recommended in order to sustain or even improve the current level of utilisation in the town.

## Abstrait

**Prédictifs de l'accouchement institutionnel dans la ville de Sodo, le Sud de l’Éthiopie**

**Contexte:**

Les femmes sont plus susceptibles de mourir pendant ou suite à l'accouchement que pendant la grossesse, mais le recours aux services d'accouchement aussi bien qu'aux soins post-partum est faible.

**Objectif:**

Déterminer la prévalence et les facteurs prédictifs de l'accouchement institutionnel dans la ville de Wolaita Sodo (ou Sodo) dans le Sud de l’Éthiopie.

**Méthodes:**

Une étude transversale a été utilisée pour observer 844 femmes ayant accouché au cours des cinq dernières années à Sodo. L’étude a utilisé un modèle d’échantillonnage à plusieurs degrés. Des codes ont été donnés à toutes les femmes identifiées dans les *kebeles* (quartiers) choisis et une technique d’échantillonnage aléatoire simple a été utilisée après génération de nombres aléatoires avec Statistical Package for Social Sciences (SPSS). SPSS a été ensuite utilisé pour effectuer des régressions logistiques binaires et multinomiales. Un IC de 95% du taux de probabilité a été appliqué pour juger de la présence de relations entre les variables.

**Résultats:**

La prévalence du recours au service d'accouchement à Sodo était de 62,2%. Le niveau d’étude du mari, la parité, le nombre de visites cliniques anténatales, l'opinion sur la qualité des soins et les connaissances concernant les signes de danger de la grossesse représentaient des facteurs prédictifs indépendants du recours aux services d'accouchement.

**Conclusion:**

Le recours au service d'accouchement institutionnel à Sodo était bien plus élevé que la moyenne nationale. Les conclusions de cette étude ont montré que la promotion des soins anténatals, l'implication des hommes dans les soins maternels, l’éducation à la santé concernant les signes de danger de la grossesse et l'amélioration de la qualité du service sont recommandées afin de maintenir ou même améliorer le niveau actuel d'utilisation dans la ville.

## Introduction

‘Pregnancy and childbirth are natural and often eventful processes, many women are at risk for developing complications during pregnancy and childbirth’.^1^ Complications related to pregnancy and delivery are the main cause of mortality and morbidity amongst women of childbearing age in third-world countries.^1^

According to the World Health Organization (WHO), maternal death is defined as:

the death of a woman whilst pregnant or within 42 days of termination of pregnancy, irrespective of the duration and site of the pregnancy, from any cause related to or aggravated by the pregnancy or its management but not from accidental or incidental causes.^2, 3^

Improved delivery service can minimise the mortality and morbidity of both the mother and the baby, because the proportion of babies delivered in a safe and clean environment (health institution) under the supervision of health professionals will increase.^4^

Institutional delivery is when childbirth happens with skilled assistance in an institution which is built, equipped and managed in order to provide a delivery service as one of its functions. The advantage of institutional delivery is that there is a greater certainty that the pregnant women will be able to access all the relevant services much easier than if she had received skilled assistance at home.^5^ In addition, identification of problems would be faster during an institutional delivery since it is carried out in an equipped healthcare setting.^6^

The majorities of births in sub-Saharan Africa still occur at home or in other non-healthcare settings. Home delivery is frequently the cheapest alternative, though it is linked highly with the risks of infection during delivery and an absence of essential equipment for any complications that might arise.^7^

In general, around 10% of women in Ethiopia give birth at a health facility. In terms of place of residence, 50% of births to urban mothers and 4% of births to rural mothers take place at a health institution.^8^ The Ethiopian government is devoted to enhancing delivery in health facilities through an integrated plan at all levels and to implementing key globally-acknowledged initiatives. However, studies exploring determinants of institutional delivery, particularly in the southern part of the country, are scarce.

## Significance of the study

Health professional-assisted delivery during all births is a key intervention for ensuring safe motherhood and is generally used as an important proxy indicator in monitoring a country's progress toward Millennium Development Goal 5. According to the 2011 Ethiopia Demographic and Health Survey report, the maternal mortality ratio (MMR) was 676 per 100 000 live births.^8^ The Ethiopian government has thus set an objective of decreasing the MMR to 267 per 100 000 live births by 2015. It is therefore essential to know which factors either hinder or facilitate the utilisation of a delivery service.

It is hoped that the provision of such vital information would inform better strategy in the bridging and/or elimination of the barriers that stand in the way of accessing institutional delivery. This would, in turn, contribute significantly to a reduction in pregnancy complications at birth, thereby reducing maternal deaths.

## Research method and design

### Participants

The source population was all women of child-bearing age who had given birth during the previous five years in Sodo town. The study population included selected women of reproductive age group who had given birth *at least once* during the previous five years.

The number of study participants was estimated by applying a single population proportion formula with the following assumptions: α = the risk of rejecting the null hypothesis (0.05), *d* = degree of precision or margin of error, *Z* = the standard score corresponding to a 95% confidence interval and *p* = 50% (the proportion of institutional deliveries).^8^ The final sample size was obtained after adding 10% to compensate for possible non-response and multiplying the result by a factor of two for design effect, resulting in a sample size of 844.

The study employed a multistage sampling scheme. First, approximately 40% (four out of 11 *kebeles*) of the town were selected randomly to conduct a census for the identification of mothers who met the study criteria. This generated a sample frame comprising 1573 women. The probability of being included in the sample was equal for all the women in the sample frame since random numbers generated using SPSS version 16 were applied in order to identify the study participants. The women who were included in the sample were interviewed at home, using a questionnaire administered in Wolaitigna (the local language).

### Design and setting

A cross-sectional study design was used in Sodo town, which is 327 km south of Addis Ababa, the capital city. In the town there are two hospitals (private and government), three health centres, seven private clinics, three pharmacies and eight drug vendors.

### Procedures

A pre-tested interviewer-administered structured questionnaire adapted from the Ethiopian Demography and Health Survey (EDHS) tool and related theses was used.^4, 9, 10^ Scales combining multiple items were used in order to measure perceived access to and quality of care at the nearest health facility.

### Data analysis

Data were checked for completeness and then coded and entered in to EpiData version 3.5.3. The data were then exported into SPSS version 16 for further analysis. Variables with significant association during bivariate analyses were entered into multiple logistic-regression models so as to control for possible confounding effects of the variables and to identify the independent predictors of institutional delivery.

The strength of association was estimated by calculating the odds ratios (OR) with 95% confidence intervals (CI). A *p*-value of < 0.05 was taken as being of statistical significance for all analyses.

## Results

### Characteristics of the respondents

A total of 810 women of child-bearing age who had given birth at least once during the previous five years participated in the study, giving a response rate of 96%. The median age of the women was 26 years (SD = 6.38). Most (743; 91.7%) of the women were married and 556 (68.6%) of the respondents belong to the Wolaita ethnic group. The majority of the women were Christian and 365 (45.1%) of the participants had a secondary and/or higher education (Table 1).

**TABLE 1:** Sociodemographic characteristics of study women (*n* = 810) Wolaita Sodo town, southern Ethiopia, 2012.

Variable	Frequency (*n*)	Percentage (%)
***Age***		
≤ 20	25	3.1
21 – 35	698	86.2
> 35	87	10.7
Median	26	–
**Marital status**		
Married	743	91.7
Divorced	39	4.8
Widowed	28	3.5
**Education status of the mother**		
No formal education	125	15.4
Primary level education	320	39.5
Secondary and above	365	45.1
**Education status of the husband**		
No formal education	59	7.9
Primary level education	262	35.3
Secondary and above	422	56.8
**Religion**		
Orthodox Christianity	324	40.0
Muslim	53	6.5
Protestant	394	48.6
Catholic	39	4.8
**Ethnicity**		
Wolaita	556	68.6
Gammo	48	5.9
Amhara	95	11.7
Oromo	36	4.4
Gurage	45	5.6
Others†	30	3.7

† Tigre, Silte, Hadiya

Looking at the obstetric history of the women, 534 (65.9%) of the women were older than 18 years when they married. The most-recent pregnancy was not planned by 217 (26.8%) of the mothers, 730 (90.1%) of them had received antenatal care at least once and 588 (72.6%) had gone for four or more visits. Of the study participants, 504 (62.2%) gave birth at a health institution during their last pregnancy. Concerning parity, 411 (50.7%) of the respondents had given birth 2 – 4 times, whilst 97 (12%) women had given birth more than five times. Well over half (555; 68.8%) of the women in this study had made their own decision as to where to deliver (Table 2).

**TABLE 2:** Obstetric characteristics of the study participants (*n* = 810), Wolaita Sodo town, Northern Ethiopia, 2012.

Variable	Frequency (*n*)	Percentage (%)
**Age at first marriage**		
< 18	276	34.1
≥ 18	534	65.9
**Parity**		
1	302	37.3
2 – 4	411	50.7
≥ 5	97	12.0
**Planned pregnancy**		
Yes	593	73.2
No	217	26.8
**Antenatal care visit**		
Yes	730	90.1
No	80	9.9
**Number of antenatal care visits**		
1	86	10.6
2 – 3	136	16.8
≥ 4	588	72.6
**Woman's decision**		
Self	557	68.8
Other†	253	31.2

† Husband, Relatives, Religious leader

### Pregnancy, danger signs, benefits of delivering in health facility

Of the total respondents, 370 (45.7%) knew at least one of the pregnancy risks and were able to name correctly one of the danger signs during pregnancy. At least three danger signs could be listed by 209 (25.8%) of the women, who were thus labeled as having good knowledge. At least one critical danger sign during labour could be named by 516 (63.7%) of the respondents, whilst 236 (29.1%) mentioned three or more danger signs, implying that they were knowledgeable in this regard. Moreover, the majority of the respondents (780; 96.3%) mentioned at least one benefit of giving birth at a health facility. Eight in 10 of the women were labeled as knowledgeable since they were able to mention at least three or more benefits of institutional delivery (Table 3).

**TABLE 3:** Awareness about danger signs in pregnancy and labour and benefits of delivering in the health facility, Wolaita Sodo town, Southern, Ethiopia, 2012 (*n* = 810).

Variables	Frequency (*n*)	Percentage (%)
**Danger signs in pregnancy**		
Yes	209	25.8
No	601	74.2
**Danger signs during labour**		
Yes	236	29.1
No	574	70.9
**Benefit of giving birth at health facility**		
Yes	650	80.2
No	160	19.8

### Perceived access to delivery care at the nearest health facility

Most respondents had favourable perceptions of the distance and travel time to, as well as the operating hours of, the nearest health facility. Health professionals at the nearest facility were reported to be available by 420 (51.9%) of respondents. In addition, 445 (54.9%) of women found the fees charged for pregnant women to be affordable (Table 4).

**TABLE 4:** Perceived access to delivery care at the nearest health facility, Wolaita Sodo town southern Ethiopia, 2012 (*n* = 810).

Variables	Frequency (*n*)	Percentage (%)
**Distance from home to the nearest health facility**		
Very far	70	8.6
Moderately far	326	40.2
Short and/or Not far	414	51.1
**Time to travel to the nearest health facility**		
Long	67	8.3
Quite long	311	38.4
Short	432	53.3
**Fee charged to pregnant women**		
Not affordable	96	11.9
Fairly affordable	269	33.2
Affordable	445	54.9
**Opening hour at the nearest health facility**		
Not suitable	109	13.4
Moderately and/or fairly suitable	212	26.2
Suitable	489	60.4
**Availability of health professional**		
Not available	114	14.1
Moderately and/or fairly available	276	34.1
Available	420	51.8

### Perceived quality of delivery care at the nearest health facility

Nearly half (360; 44.4%) of the women perceived that the facilities in the delivery room were suitable. Similarly, most of the respondents were happy with staff integrity, staff respectfulness toward pregnant women, the health professionals’ ability to examine their patients and the keeping of patient privacy during examination. More than six in 10 of the women felt that the health professionals were not willing to communicate with mothers during delivery (Table 5).

**TABLE 5:** Perceived quality of delivery care at the nearest health facility, Wolaita Sodo town Southern Ethiopia, 2012 (*n* = 810).

Perceived quality of healthcare	Frequency (*n*)	Percentage (%)		
**Staff integrity at the nearest health facility**				
Not honest	95	11.7		
Fairly honest	284	35.1		
Honest	392	48.4		
I don't know	39	4.8		
**Health professionals’ competence**				
Not capable	435	53.7		
Fairly capable	267	33.0		
Capable	67	8.3		
I don't know	41	5.1		
**Patients’ access to drugs**				
With difficulty	99	12.2		
With relative ease	287	35.4		
With ease	357	44.1		
I don't know	67	8.3		
**Adequacy of equipment to provide delivery care**				
Inadequate	115	14.2		
More or less adequate	210	25.9		
Adequate	356	44.0		
I don't know	129	15.9		
**Examination and/or delivery rooms’ conditions**				
Inadequate	171	21.1		
More or less adequate	215	26.5		
Adequate	340	42.0		
I don't know	84	10.4		
**Health professionals’ ability to examine pregnant women**				
Not very well	54	6.7		
Moderately and/or fairly well	230	28.4		
Well	474	58.5		
I don't know	52	6.4		
**Health professionals’ openness with pregnant women**				
Not very open	499	61.6		
Fairly open	168	20.7		
Open	73	9.0		
I don't know	70	8.6		
**Health professionals’ respectfulness toward pregnant women**				
Not very respectful	93	11.5
Fairly respectful	237	29.3		
Respectful	443	54.0		
I don't know	37	4.6		
**Health professionals’ time devoted to patients**				
Inadequate	90	11.1		
More or less adequate	371	45.8		
Adequate	297	36.7		
I don't know	52	6.4		
**Given privacy for patient during examination**				
Inadequate	124	15.3		
More or less adequate	189	25.3		
Adequate	450	55.6		
I don't know	47	5.8		
**Number of health professionals**				
Inadequate	171	21.1		
More or less adequate	235	29.0		
Adequate	335	41.4		
I don't know	69	8.5		
**Suitability of facilities at the delivery rooms**				
Not well suited	80	9.9		
Relatively well suited	255	31.5		
Well suited	360	44.4		
I don't know	115	14.2		

### Factors affecting birth in the health facility

In the first model, of all the sociodemographic variables entered in the model, maternal age, maternal education, family income, husband occupation and husband education were found to have a statistically-significant association with place of delivery (*p* < 0.05). In line with these findings, mothers older than 35 years were 44.1% less likely to give birth at health facilities compared with those between 20 and 35 years of age (crude odds ratio [COR] = 0.559; 95% CI = 0.357–0.875). Mothers whose husbands had never had any formal education were 80.3% less likely to deliver at a health facility than those whose husbands had completed secondary level and above (COR = 0.197; 95% CI = 0.111–0.351). Women with no formal education were 70.4% less likely to use an institutional delivery service than women who had received secondary and above education (COR = 0.296; 95% CI = 0.195–0.452). Furthermore, women whose husbands were merchants or daily labourers were less likely to utilise health facilities during delivery compared with those women whose husbands were government employees (COR = 0.600; 95% CI = 0.385–0.935 and COR = 0.388; 95% CI = 0.249–0.605 respectively).

Amongst the obstetric variables entered in the first model, parity, frequency of antenatal care and pregnancy planning were associated significantly with place of delivery (*p* < 0.05).

### Perceived access to care, perceived quality and knowledge

Perceived access to care, perceived quality of care, maternal knowledge regarding the benefits of giving birth at a health facility, danger signs during pregnancy and labour were associated significantly with the place of delivery (*p* < 0.05). A 10-unit increment in the score of *perceived access* increased the odds of utilisation of an institutional delivery service by 13 (COR = 1.344; 95% CI = 0.258–1.436). Similarly, a 10-unit increment in the score of *perceived quality* increased the odds of utilisation of an institutional delivery service by 12 (COR = 1.232; 95% CI = 1.192–1.273).

With regard to maternal knowledge, mothers who knew three or more danger signs of pregnancy were three times more likely to deliver at a health facility than mothers who had little or no knowledge thereof (COR = 3.004; 95% CI = 2.071–4.358). Similarly, mothers who knew three or more danger signs of labour were twice as likely to give birth at a health facility than those who were unable to mention any (COR = 2.136; 95% CI = 1.528–2.987). Likewise, women who knew three or more benefits of facility-based delivery were more than twice as likely to deliver in a health facility than women who knew of less or no benefits thereof (COR = 2.421; 95% CI = 1.703–3.436).

### Independent predictors of place of delivery

Husband educational status, number of antenatal care visits, parity, knowledge regarding pregnancy danger signs and perceived quality of care were independent predictors of place of delivery (Table 6).

**TABLE 6:** Independent predictors of institutional delivery service utilisation in Wolaita Sodo town, southern Ethiopia.

Variables	Place of delivery			

	Home (*n* = 306)	Health facility (*n* = 504)	Crude Odds Ratio	95% Confidence Interval
**Parity**				
1	93	209	1.601 (1.171–2.190)	2.234 (1.461–3.415)†
2 – 4	171	240	1.00	–
≥ 5	42	55	0.933 (0.597–1.459)	1.680 (0.897–3.147)
**Planned pregnancy**				
Yes	204	389	1.692 (1.233–2.320)	0.819 (0.511–1.312)
No	102	115	1.00	–
**Antenatal care frequency**				
≤ 1	62	24	0.161 (0.098–0.267)	0.320 (0.165–0.622)†
2 – 3	71	65	0.382 (0.261–0.558)	0.522 (0.316–0.861)†
≥ 4	173	415	1.00	–
**Knowledge about danger signs of pregnancy**				
Yes	43	166	3.004 (2.071–4.358)	2.134 (1.334–3.414)†
No	263	338	1.00	–
**Knowledge about danger signs of labour**				
Yes	61	175	2.136 (1.528–2.987)	1.487 (0.901–2.453)
No	245	329	1.00	–
**Access and quality to care**				
Perceived access to care	–	–	1.344 (1.258–1.436)	0.987 (0.895–1.087)
Perceived quality of care	–	–	1.232 (1.192–1.273)	1.217 (1.174–1.261)†
**Age of the respondent**				
≤ 20	8	17	1.216 (0.517–2.857)	1.218 (0.319–4.653)
20 – 35	254	444	1.00	–
> 35	44	43	0.559 (0.357–0.875)	0.753 (0.354–1.604)
**Education status of the mother**				
No formal education	70	55	0.296 (0.195–0.452)	0.888 (0.449–1.757)
Primary level education	136	184	0.511 (0.371–0.703)	1.112 (0.684–1.808)
Secondary and above	100	265	1.00	–
**Education status of the husband**				
No formal education	38	21	0.197 (0.111–0.351)	0.248 (0.116–0.530)†
Primary level education	119	143	0.429 (0.310–0.594)	0.447 (0.298–0.670)†
Secondary and above	111	311	1.00	–
**Occupation of the husband**				
Government employee	49	139	1.00	–
Private employee	45	104	0.815 (0.505–1.314)	0.834 (0.436–1.597)
Merchant	67	114	0.600 (0.385–0.935)	0.660 (0.350–1.242)
Daily labourer	80	88	0.388 (0.249–0.605)	0.765 (0.389–1.506)
Farmer	7	6	0.302 (0.097–0.943)	0.462 (0.084–2.539)
Other‡	20	24	0.423 (0.215–0.832)	0.338 (0.136–0.844)
**Monthly income**				
≤ 500	258	380	1.00	–
501 – 999	20	40	1.358 (0.776–2.376)	0.974 (0.449–2.116)
≥ 1000	28	84	2.037 (1.291–3.214)	1.256 (0.644–2.449)

† Significant at 95%.

‡ Carpenter, weaver.

The effect of perceived quality of care on delivery at a health facility was reduced substantially from the bivariate to the multivariate models; however, women who perceived quality of care to be high had a significantly higher chance of delivering at a health facility than women who perceived quality of care to be low. A 10-unit increment in the score of perceived quality of care increased the odds of facility-based delivery by 12 (adjusted odds ratio [AOR] = 1.217; 95% CI = 1.174–1.261).

The effect of a woman's level of education on delivery at health facilities did not have any statistical significance but the husband's educational level had a significant and favourable effect on the place of delivery: women whose partners had completed only primary school were 55.0% less likely to deliver at a health facility than women whose partners had passed secondary school and higher (AOR = 0.447; 95% CI = 0.298–0.670). In addition, women whose partners had never had any formal education were 75.0% less likely to give birth at a health facility than women whose partners had passed secondary school and higher (AOR = 0.248; 95% CI = 0.116–0.530).

Those women who were giving birth for the first time had a greater chance of delivering in a health institution as compared with women who had given birth previously (AOR = 2.234; 95% CI 1.461–3.415). Women who had been for only one antenatal care visit were 68.0% less likely to deliver at a health facility than women who had been for four or more visits (AOR = 0.320; 95% CL = 0.165–0.622).

With regard to maternal knowledge, mothers who knew at least three danger sign of pregnancy were twice as likely to deliver at a health facility than mothers who did not (AOR = 2.134; 95% CI = 1.334–3.414). By contrast, factors such as whether the pregnancy was wanted or not, age of the respondent, partner occupation, maternal education, income, perceived access, knowledge of the danger signs of labour and benefits of giving a birth in a health facility were not associated statistically with the place of delivery.

## Ethical considerations

Ethical clearance to undertake the study was obtained from Jimma University, College of Public Health and Medical Science (Ref No: RPGC/69/2012). The Sodo town health office was also contacted regarding implementation of the study. A consent form was approved prior to the interviews and the consent, when signed by the women selected for the study, addressed issues relating to confidentiality and autonomy.

## Trustworthiness

The study design and procedures we followed were systematic and acceptable scientifically. The sampling process applied to choose participants was random. Random numbers generated using SPSS were applied for identification of the study participants. Data were cleaned, then univariate, bivariate and, finally, multiple logistic analysis was carried out in order to control (adjust) for possible confounding variables.

## Discussion

The level of institutional delivery service utilisation in the current study was 62.2%. Although delivery services were available, four out of every 10 women in this study gave birth at home in the absence of any assistance by a trained professional. This finding is consistent with the findings for Entebbe, Uganda (63%) and the EDHS 2011 report (50%).^8, 11^ However, the level of delivery service utilisation in this study is less than that of Addis Ababa where 82.3% of the women used institutional delivery services.^8^ This might be related to the fact that the population in Addis Ababa enjoys a better access to and quality of delivery care compared with other urban areas in the country.

Those women who had one child were twice as likely to deliver in a health institution compared with women having 2–4 children. This is consistent with the nationwide Demography and Health Survey result and a report from Gondar (a town in northern Ethiopia) where delivery in a health facility was observed to be more frequent amongst mothers for their first birth.^4, 12^ It has been argued that women fear complications or lack confidence in the face of problems during the first pregnancy which leads to a preference to deliver in a health facility, in search of better protection. However, as the birth order increases mothers tend to rely on past experience and underestimate the risks associated with the upcoming birth. It is also likely that as the family size increases, fewer resources are available, preventing them from seeking attention at a health facility.^13, 14^

Use of antenatal care was associated with an increased probability of institutional delivery. Women who had been for four or more antenatal care visits per pregnancy had a significantly-lower chance of delivering at home. Similar findings were reported by the Ethiopian DHS^4^ and skilled birth attendance increased with more antenatal care visits, as reported by Mpembeni et al. from Tanzania.^15^ These findings corroborate the WHO as well as the Ethiopian Ministry of Health's recommendations that every pregnant woman should have at least four standard antenatal care visits during a pregnancy.^16^ This is expected because during an antenatal care visit women are provided with health education and information about the benefits of giving a birth in the health facility for the child as well as for the mother's health. Given this finding, it is evident that promoting increased antenatal care uptake could be used as an effective strategy to increase the proportion of births occurring in the health-facility setting.^16^

Findings in this study implied that women who reported low perceived quality of services had a lower probability of utilising a health facility-based delivery service. It has been argued that women are interested in the appearance of the health facility in the way in which the healthcare providers handle mothers in labour. The feature of provider–client interaction is a critical issue in this regard and unwelcoming behaviour from the healthcare worker is a serious barrier to the use of health services.^10, 17^

Maternal knowledge regarding pregnancy warning signs was found to be an independent predictor of giving birth in a health-facility setting. This is consistent with the finding from a study in India which identified ‘lack of recognition of perceived seriousness of health problems’ as being a significant factor for not preferring delivery in a health facility, even though half of the maternal deaths observed were related to non-use of this service.^18^ Similarly, a study from Tanzania indicated a nearly threefold increase in skilled birth attendance when accompanied by better knowledge about risks in pregnancy.^15^ This might imply that mothers who know the danger signs have a greater fear of complications, compelling them to seek skilled attendance during birth. Moreover, maternal awareness about the warning signs in pregnancy and labour initiates quick and correct decisions regarding the use of delivery services.

In general, the results of this study suggested that women with better-educated husbands were more likely to use a health facility during delivery than women who have husbands with no education or only primary school education. Similar findings were reported by Shariff and Singh from India.^19^

Findings in this study should be interpreted in the light of the inherent limitations of the study. Recall bias was a possibility since the women were inquired about events which occurred during a five-year period. However, the questioning was focused on the most recent experience of delivery in order to minimise this possibility.

## Conclusions

The proportion of women who used institutional delivery service in the study area was relatively promising. The independent predictors of institutional delivery service utilisation in this study were the number of antenatal care visits, parity, partner educational status, the woman's knowledge about danger signs in pregnancy and perceived quality of delivery care.

## Recommendations

The provision of tailored messages to all members of the community regarding the risks of pregnancy, danger signs of labour and benefits of giving birth at the health facilities would appear to be a very important intervention in increasing utilisation of delivery care at the health facilities. Promotion of early uptake of antenatal care visits and the completion of four standard visits by pregnant women is also recommended. Hence, linking antenatal care and delivery care services and enhancing male involvement in maternal healthcare is also advisable. Further research should be conducted in order to explore the relationship between quality of delivery care services and utilisation of these services by pregnant women.
